# High efficient OLED displays prepared with the air-gapped bridges on quantum dot patterns for optical recycling

**DOI:** 10.1038/srep43063

**Published:** 2017-02-17

**Authors:** Hyo-Jun Kim, Min-Ho Shin, Joo-Suc Kim, Se-Eun Kim, Young-Joo Kim

**Affiliations:** 1 Yonsei University, Department of Mechanical Engineering, Seoul, 120-749, Korea

## Abstract

An optically efficient structure was proposed and fabricated to realize high brightness organic light emitting diode (OLED) displays based on a white OLED prepared with the air-gapped bridges on the quantum dot (QD) patterns. Compared with a conventional white OLED display, in our experiments, the optical intensity of the proposed OLED display shows the enhancement of 58.2% in the red color and 16.8% in the green color after applying the air-gapped bridge structure on QD patterns of 20 wt% concentration. This enhancement comes from the two facts that the QD patterns downconvert unnecessary blue or blue/green light to the required green or red light and the air-gapped bridges increase the color conversion efficiency of QDs by optical recycling using total internal reflection (TIR) at the interface. In addition, the color gamut of the proposed OLED display increases from 65.5 to 75.9% (NTSC x, y ratio) due to the narrow emission spectra of QDs.

Flat panel display (FPD) based on organic light emitting diodes (OLEDs) has attracted much attention in both scientific and industrial communities as a strong candidate to replace liquid crystal displays (LCDs) due to its advantages of self-emitting structure, thin thickness, wide viewing angle, high contrast ratio, and short response time^1^,^2^,^3^,^4^,^5^. However, individual red, green, and blue (RGB) pixel based OLEDs have technical limitations in realizing large-sized displays with high resolution, because the fine metal mask (FMM) required for patterning an RGB OLED still has some serious technical barriers such as inaccurate precision alignment and contact with glass substrate^6^,^7^,^8^,^9^. To overcome these barriers, a method using white OLEDs with color filter (CF) has emerged as an alternative OLED display structure. This white OLED based display has some advantages for manufacturing large-sized and high-resolution displays at low cost with high yield in mass production since it does not require the FMM for the RGB pixel patterning^10^,^11^,^12^,^13^. However, the optical efficiency (or power efficiency) of the white OLED based display is too low since the usage of CF results in the inevitable absorption loss of light over the unmatched color range from white OLED. A lot of works have attempted to solve the issue of high power consumption in white OLED displays, including novel tandem structure consisting of multiple electroluminescent units connected electrically in series^14^,^15^,^16^,^17^,^18^,^19^, top emission white OLEDs allowing a high aperture pixel ratio^20^,^21^,^22^,^23^,^24^,^25^,^26^, and a RGBW pixel pattern with extra highly efficient white subpixels^27^,^28^,^29^,^30^. However, these attempts are still not enough to solve the fundamental problem, i.e., the absorption loss by the CF over the unmatched color range.

Recently, we proposed and published a new approach to improve the optical efficiency in red color range of bottom emitting white OLED display by applying the quantum dot (QD) patterned thin film to enhance the emission light from red QD dispersed in photoresist (PR), as shown in Fig. 1(a). By downconverting the unnecessary light from blue and green to red color, this structure reduces the absorption loss by the CF and resulted in the improved optical intensity of red light by 40.2%^31^. However, there are still several points of improvement for further enhancement of optical intensity. The first point of improvement is related to the absorption loss by the red CF. This absorption loss by the red CF was measured experimentally and shown in Fig. 1(b), as a yellow area, which means the difference between the intensities of the white OLEDs with only red QD film and with both red QD film and red CF. Since red QD film has a relatively thin thickness of 2 μm, all green and blue light cannot be absorbed completely, as shown in Fig. 1(c). Thus some of the green and blue light from white OLED passes through the red QD film to be an absorption loss by the red CF. The second point of improvement is related to the absorption loss by the other CFs of blue and green color located near the red CF. When red light has larger incident angle than the critical angle between bottom glass and the air, it is reflected at the surface of the bottom glass, as described in Fig. 1(c). Since the bottom glass is usually thick of around 300 μm thickness, most of this reflected light reaches to its neighbor CFs due to the relatively narrow width of subpixels with around 40 μm, resulting in low extraction efficiency. Both types of light absorption loss also occur in the green subpixel. Thus, we think about an additional effective component by optical recycling to overcome both absorption loss points and improve the optical intensity of white OLED display.

In this study, we have applied not only both red and green QD patterns as color downconverting components but also an additional optical recycling component of air-gapped bridges to improve the optical efficiency of white OLED displays. In addition, we have developed the improved method for patterning of QD patterns by photolithography to improve the quantum efficiency of QDs in the patterned QD film. Thus the QD patterns show a key function to downconvert the unnecessary light from the white OLED effectively that would be cut by the CFs, while the air-gapped bridges has a key function to recycle the light to the QDs to be absorbed and downconverted again to the required color for the better brightness of OLED displays.

## Results

### Mechanism of proposed white OLED structure

**Figure 2:**
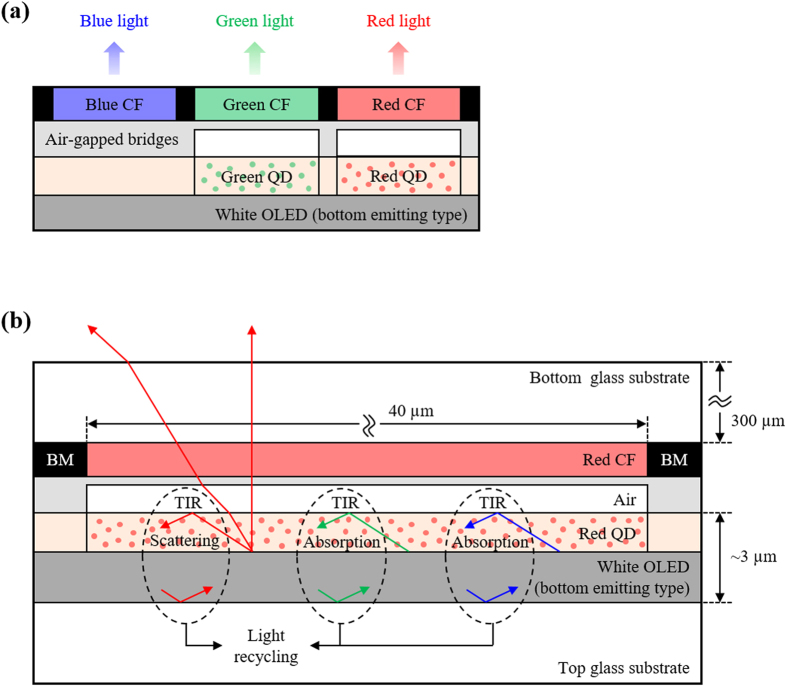
(**a**) Schematic illustration of new white OLED structure prepared with the air-gapped bridges on the QD patterns proposed in this study and (**b**) mechanism of optical intensity enhancement in red subpixel with air-gapped bridges on QD patterns.

As shown in Fig. 2(a), the air-gapped bridges are inserted between the CFs and the white OLED after being aligned on the red and green QD patterns. The mechanism for the red subpixel in the proposed design structure is explained in Fig. 2(b). At the interface between the red QD patterns (refractive index of 1.65) and air-gapped bridges (refractive index of 1.0), the critical angle is about 37.3°. Some of the green and blue light from the white OLED is absorbed by the red QDs and downconverted to the required red light, resulting in the enhanced red optical intensity. The green and blue light unabsorbed by the red QDs will be cut by the red CF as an absorption loss, because total internal reflection does not occur at the interface between the red QD patterns and red CFs. By adding air-gapped bridges, however, the incident light over a critical angle can be reflected by total internal reflection (TIR) between the red QD patterns and air-gapped bridges. Then, some of the reflected light is absorbed again through the red QD patterns while some is reflected by metal electrode of the white OLED. Therefore the downconverting by the red QDs increases by the optical recycling between the air-gapped bridges and metal electrode of the white OLED. This is a solution for the absorption loss by the red CF mentioned above as a first point of improvement.

In addition, TIR does not occur at the surface of bottom glass, because only red light of incident angle below the critical angle between air-gapped bridges and red QD patterns can pass through the air-gapped bridges. Since red color of the reflected light at the surface of red QD patterns is scattered by red QDs with diverse direction, the red light having an incident angle below the critical angle increases to improve the extraction efficiency more effectively, which is a solution for the absorption loss by the other CFs of blue and green color located near the red CF mentioned as a second point of improvement. The scattering characteristics of light in the QD film were measured and analyzed to understand the effect of air-gapped bridges to improve the extraction efficiency in our proposed white OLED structure (see Supplementary Information with Figs S1 and S2).

### Preparation of core components

To realize the proposed white OLED structure, we fabricated core components and set up the measurement system using a bottom emitting white OLED, a CF array, QD patterns, and air-gapped bridges. A conventional white OLED composed with a glass substrate, transparent anode, tandem emitting layer (two peaks at 455 and 555 nm wavelength), and aluminum as a cathode without thin film transistor (TFT) circuitry was selected as a white light source, while a CF array with an RGB subpixel size of 40 × 115 μm was used.

**Figure 3:**
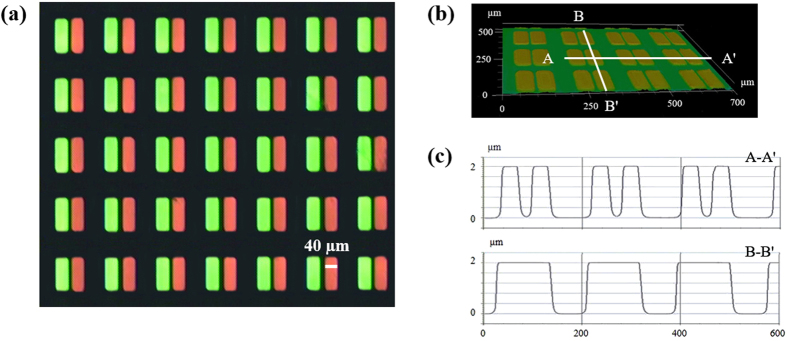
(**a**) Fluorescence microscope image of QD patterns prepared with 20 wt% concentration and exited by a light source of 405 nm wavelength, (**b**) three-dimensional image of QD patterns, and (**c**) height profiles of cross sections indicated by the solid line in (**b**).

To apply the QDs to the white OLED display, it is important to fabricate a thin film of both red and green QD patterns on the same substrate while keeping high concentration of QDs. In this experiment, PR dispersed with QDs in a solvent of propylene glycol monomethyl ether acetate (PGMEA) was used to fabricate the red and green QD patterns by repeating the photolithography process twice. Figure 3(a) shows a fluorescence microscope (Olympus, IX71) image for the QD patterns prepared with 20 wt% concentration and exited by a light source of 405 nm wavelength. A single QD pattern has a size of 40 × 115 μm (the same size as a subpixel of the CF array) as shown in Fig. 3(b) taken by 3D laser scanning microscope (KEYENCE, VK-X200 K) while its thickness was controlled as 2 μm as shown in Fig. 3(c) taken by stylus profiler (Bruker, Dektak XT). In addition, the quantum efficiency of QDs in the film was measured for the separately prepared red and green QD films with same conditions using a quantum efficiency analysis system (OTSUKA, QE-1000). The quantum efficiency of red and green QDs in the film prepared with 20 wt% concentration resulted in 56 and 45%, respectively. These values are higher than those in previous research that used a negative PR and solvent of cyclopentanone for QD patterning^31^. We believe that better chemical matching from the ligand of QD, solvent, and PR used in this paper results in more efficient and reliable result in the quantum efficiency of QDs.

**Figure 4:**
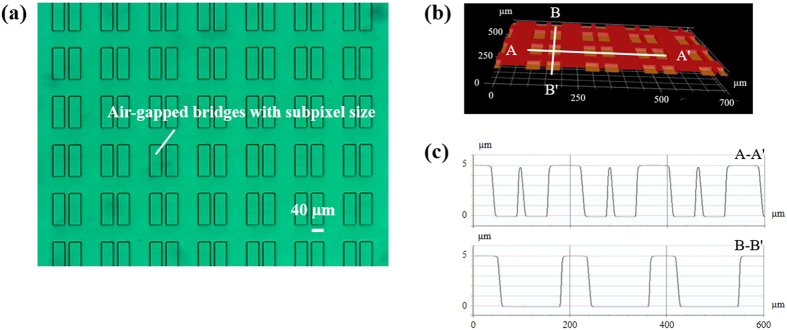
(**a**) Optical microscope image of air-gapped bridges, (**b**) three-dimensional image of air-gapped bridges, and (**c**) height profiles of cross sections indicated by the solid line in (**b**).

Air-gapped bridges were fabricated using an imprinting process to be aligned vertically with the red and green QD patterns. Figure 4(a) shows an optical microscope image for the air-gapped bridges having a same pattern with the QD patterns. A single air-gapped pattern has a size of 40 × 115 μm (same size as a subpixel of both CF array and QD pattern) and a thickness of 5 μm, as shown in Fig. 4(b) taken by 3D laser scanning microscope and Fig. 4(c) taken by stylus profiler, respectively. After preparing all core components, including a CF array, air-gapped bridge, and QD patterns with a PR coating for the flat surface, they were assembled using adhesion polymer while keeping the vertical alignment under the optical microscopy. Finally, a white OLED panel was located under the assembled core components to realize the proposed white OLED display structure.

### Improved optical intensity of proposed white OLED structure

**Figure 5:**
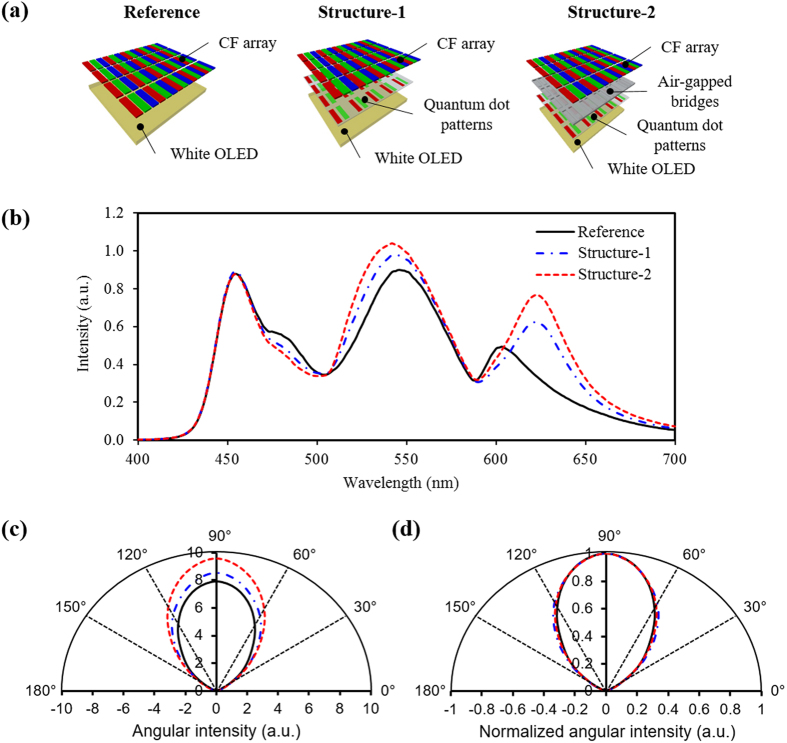
(**a**) Schematic illustration of measurement configuration for optical intensity in three white OLED structures and (**b**) optical intensity spectra, (**c**) angular intensity, and (**d**) normalized angular intensity measured from reference, structure-1, and structure-2.

To verify the enhanced optical intensity of the proposed white OLED structure, we measured the optical intensity spectra using an integrating sphere. A white OLED panel with only CF array was prepared and the optical intensity spectra was also measured as a reference, as shown in Fig. 5(a). The white OLED display of ‘structure-1’ was prepared after adding the QD patterns to the reference structure for the comparison. Finally, the white OLED display of ‘structure-2’ was prepared after adding both the air-gapped bridges and QD patterns as the proposed structure. Figure 5(b) shows optical intensity spectra measured from the reference, structure-1, and structure-2. The unnecessary light from white OLED is partially absorbed by red and green QDs to be downconverted to the light of longer wavelength for the emission of the required light. As a result, the structure-1 resulted in higher optical intensity than the reference. As explained above, air-gapped bridges cause the enhancement of absorption of the QDs by optical recycling between the surface of the QD patterns and the metal electrode of the white OLED to produce more light having a wavelength range required in the CFs. As a result, structure-2 resulted in the highest optical intensity compared to the reference and structure-1. It is also observed that the peak wavelengths of spectra in the red and green ranges were shifted after adding the QD patterns and air-gapped bridges. Since the red CF has a transmittance close to 95% after 610 nm and the green CF has its highest transmittance at around 530 nm, we selected the red QDs with 630 nm peak wavelength and green QDs with 530 nm peak wavelength to produce the QD patterns after also considering the absorption rate and full width at half maximum (FWHM) of the QDs. Thus it is understood that the peak wavelength shift such as a red-shift in the red range and a slight blue-shift in the green range, is also related to the improved excitation of required light by the QD patterns and air-gapped bridges. Figure 5(c) shows the angular intensities measured using a rotating jig and spectrometer for the three white OLED structures. As expected, the optical intensity in the white OLED increases over the entire angular range after adding the QD patterns and further increases after adding the air-gapped bridges. As shown in Fig. 5(d), the normalized angular intensities for the three white OLED structures are almost same. From this result, we can confirm that the proposed white OLED structure with the QD patterns and air-gapped bridges is little affected on the relative intensity as a function of viewing angle, compared to that of conventional white OLED display.

For more quantitative analysis, the enhanced optical intensities and luminous efficacies of the RGB subpixels for the three white OLED structures were determined by integrating the measured spectra over wavelength, as summarized in Table 1. In all cases, the white OLED panel was operated at a voltage of 8 V and a current of 8 mA. Under this operating condition, the optical intensities of the red and green subpixels in structure-1 were enhanced by 32.1 and 9.6%, respectively, compared with the reference. In addition, the optical intensities of the red and green subpixels in structure-2 were enhanced further by 58.2 and 16.8%, respectively, compared with the reference. Since the red QD is related with both the blue and green colors from the white OLED and the green QD is related with only the blue color from the white OLED, the degree of enhancement of the red subpixel is higher than that of the green subpixel. We think this result is matched well to solve the power consumption issue in white OLED displays. Since the spectra of white OLED panel currently consists of blue and yellow color distribution, the optical intensity in the red range requires more improvement compared to the green range. In addition, the luminous efficacies of the red and green subpixels were measured as 1.09 and 7.9 lm/W for the reference, 1.28 and 8.52 lm/W for the structure-1, and 1.49 and 9.69 lm/W for the structure-2. The luminous efficacy of blue subpixel was measured as 0.7 lm/W for all cases. Since the visibility of green color is higher than that of red color, the luminous efficacy of the green subpixel has a bigger impact on total luminous efficacy. In this study, we focused on not photometry units but radiometry units. Therefore, the peak wavelength of green QD emission was selected as the peak wavelength of green CF transmittance (about 530 nm) to maximize optical intensity (radiometry unit). Thus, it is possible to enhance the luminous efficacy (photometry unit) further by changing the peak wavelength of green QD emission to the peak wavelength of eye sensitivity (about 555 nm).

### Improved color gamut of proposed white OLED structure

**Figure 6:**
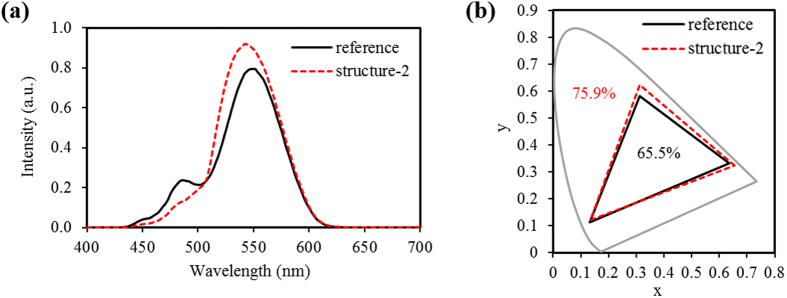
(**a**) Optical intensity spectra of green subpixel and (**b**) color gamut measured from reference and structure-2.

In white OLED displays using a commercial CF, there may be a technical limit to realizing high color gamut because a commercial CF has a relatively wide spectral transmittance to realize higher optical intensity. Figure 6(a) shows the optical intensity spectra of the green subpixel measured from the reference and structure-2. The spectra of the reference has two light peaks centered at 485 and 550 nm due to wide spectral transmittance of the green CF, and this causes reduction of color gamut in the white OLED display. This problem can be solved to some extent by applying a green QD pattern as a color converting component to the white OLED. The spectra of structure-2 has one light peak at 545 nm due to the narrow emission spectra of the green QDs after being excited with wide absorption spectra to the blue light wavelength. Figure 6(b) shows that the color gamut increases from 65.5 to 75.9% (NTSC x, y ratio) after applying air-gapped bridges on QD patterns in our experiments.

## Discussion

Air-gapped bridges aligned with the red and green QD patterns were fabricated using both general photolithography and imprinting processes and applied to a white OLED display to realize both high optical intensity and high color gamut. The QD patterns downconvert unnecessary blue or blue/green light to the required green or red light while the air-gapped bridges increase the color conversion efficiency of QDs by optical recycling using TIR at the interface. Compared with a conventional white OLED display, in our experiments, the optical intensity of the proposed OLED display shows the enhancement of 58.2% in the red range and 16.8% in the green range after applying the air-gapped bridge structure on QD patterns of 20 wt% concentration. In addition, the color gamut of the proposed white OLED display increases from 65.5 to 75.9% (NTSC x, y ratio) due to the narrow emission spectra and relatively wide absorption spectra to the blue light wavelength of the green QDs. Consequently, our proposed white OLED structure integrated with air-gapped bridges on QD patterns has an advantage in terms of improving the optical intensity as well as widening the color gamut. In addition, we believe that this combination of QD pattern as a color-converting component and air-gapped bridge as an optical-recycling component shows high potential to be applied to other optoelectronic devices such as LCDs, light-emitting diodes (LEDs), and photovoltaic cells.

## Methods

### Fabrication of QD patterns

Nonpolar ligand (oleic acid)-capped CdSe/ZnS core/shell QDs were used. First, a solvent of red QD solution was exchanged from hexane to PGMEA at a concentration of 50 mg/ml using a rotary evaporator (Heidolph, Hei-VAP), while confirming no aggregation of QDs. Then, red QDs dissolved in PGMEA were mixed with positive PR (Merck, AZ GXR-601) with QDs of 20 wt% concentration. Because the main solvent of the PR is PGMEA, red QDs dissolved in PGMEA and PR are mixed well without other mixing processes. Then, experimentally optimized photolithography processes were carried out on the glass substrate; 1) spin coating at 4000 rpm for 2 μm thickness of film, 2) pre-baking at 90 °C for 90 s, 3) exposure of i-line UV radiation with energy of 825 mJ/cm^2^ using a mask aligner (Suss Microtec, MA6), 4) immersing in developer (Merck, AZ 300 MIF) for 60 s, 5) hard baking at 110 °C for 90 s, and 6) oxygen plasma (diener, ZEPTO) for removing the residual layer. After patterning the green QDs in the same substrate by repeating the processes explained above, both red and green QD patterns were realized.

### Fabrication of air-gapped bridges

Using a photomask designed for air-gapped bridges, a Si master having 5-μm-height patterns was fabricated by photolithography process followed by reactive ion etching. Then, UV resin (Minuta technology, MINS-ERM) was dispensed on a PET substrate and imprinted using the Si master under pressure. After sufficient exposure of UV light, air-gapped bridges on the PET substrate were released from the Si master.

## Additional Information

**How to cite this article**: Kim, H.-J. *et al*. High efficient OLED displays prepared with the air-gapped bridges on quantum dot patterns for optical recycling. *Sci. Rep.* **7**, 43063; doi: 10.1038/srep43063 (2017).

**Publisher's note:** Springer Nature remains neutral with regard to jurisdictional claims in published maps and institutional affiliations.

## Supplementary Material

Supplementary Information

## Figures and Tables

**Figure 1:**
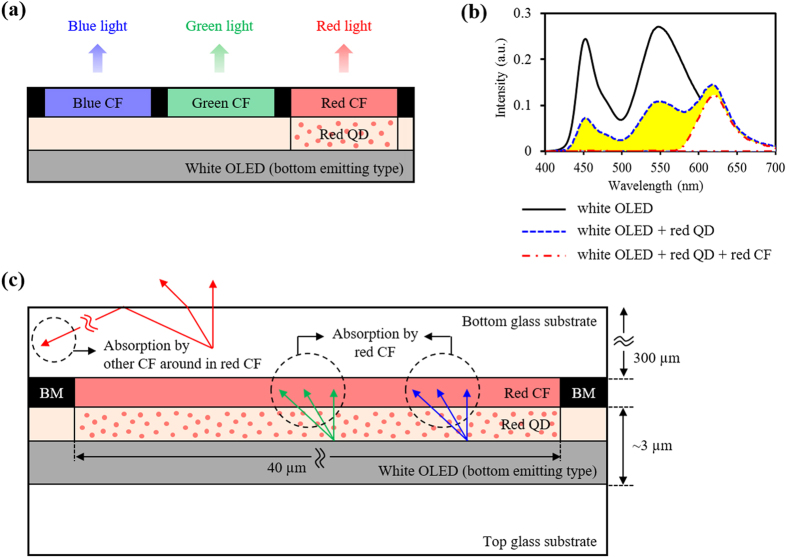
(**a**) Schematic illustration of white OLED display with red QD pattern, (**b**) the measured intensity spectra of white OLED with and without red QD and red CF, and (**c**) mechanism of light loss in white OLED display with red QD pattern.

**Table 1:** Enhanced optical intensity and luminous efficacy of white OLED displays measured from each subpixel of the reference, structure-1, and structure-2.

Device design	Enhanced optical intensity (%)		Luminous efficacy (lm/W)			
	Red subpixel	Green subpixel	Red subpixel	Green subpixel	Blue subpixel	Total
Reference	—	—	1.09	7.9	0.7	9.69
Structure-1	32.1	9.6	1.28	8.52	0.7	10.5
Structure-2	58.2	16.8	1.49	9.69	0.7	11.88
