# Supplementary material for: High efficient OLED displays prepared with the air-gapped bridges on quantum dot patterns for optical recycling
Source: Sci Rep. 2017 Feb 17;7:43063. doi: 10.1038/srep43063 (PMC5314370; doi:10.1038/srep43063)
Supplement: Supplementary Information [file srep43063-s1.pdf]

Supplementary Information

High efficient OLED displays prepared with the air-gapped bridges on quantum dot patterns for optical recycling

Hyo-Jun Kim[1], Min-Ho Shin[1], Joo-Suc Kim[1], Se-Eun Kim[1], and Young-Joo Kim[1,*]

[1]Yonsei University, Department of Mechanical Engineering, Seoul, 120-749, Korea

*yjkim40@yonsei.ac.kr

Scattering characteristics of the QD film

To evaluate the distribution of light scattering in the QD film, we made up the measurement system based on a laser, detector, spectrometer, rotating jig, glass hemisphere jig, and QD dispersed PR film with thickness of 2 µm and QD concentration of 20 wt% on glass substrate, as shown in Fig. S1(a). The incident light from a laser is entered into the QD film with a vertical direction and the detector can

analyze the light scattering as a function of scattering angles. The red or green QD film was measured with a red laser (a central wavelength of 635 nm) or green laser (a central wavelength of 520 nm), respectively. The scattering light to both the forward and backward directions were measured and analyzed. Figure S1(b) shows the normalized light scattering distribution obtained from the red and green QD film as a function of angle from 0 to 360 degree. Both the red and green QD film have similar distribution of relatively large portion to the forward scattering and relatively small portion to the backward scattering. Also, the scattered light has an angle distribution from 60 to 120 degree in the forward direction and from 240 to 300 degree in the backward direction, respectively.

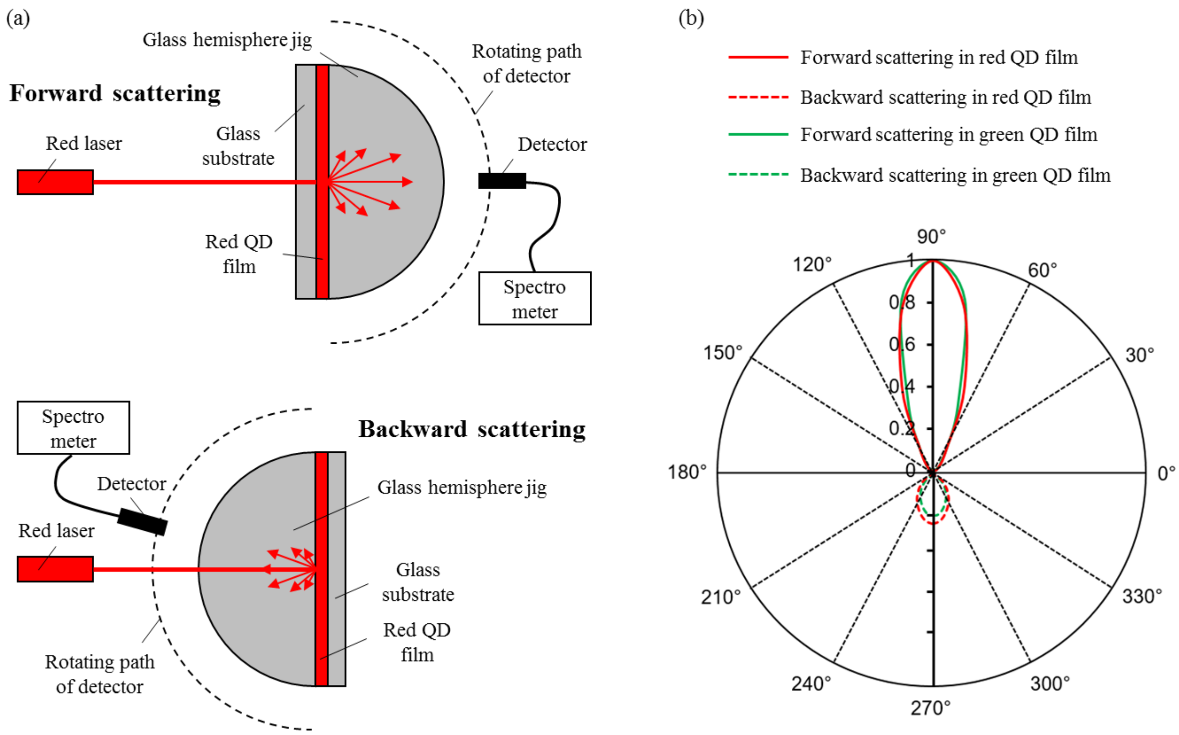

Figure S1. (a) Schematic illustration of measurement system for light scattering in QD film and (b) normalized light scattering distribution measured from the red and green QD film.

To evaluate the amount of scattered light in the QD film, we measured vertical transmittance of the red and green QD film using a spectrophotometer (U-3900, HITACHI), as shown in Fig. S2. From this graph, we can understand that around 25% of red incident light is scattered in the red QD film, since only transmittance and scattering occur in the red QD film with a negligible absorption of red light in red range. Similarly, about 35% of green incident light is scattered in the green QD film. Thus we can confirm that the reflected light on the surface of QD patterns with the air-gapped bridges can effectively scattered inside the QD film to be changed the propagation direction to be escape from the QD film for the enhanced extraction efficiency in our proposed white OLED display structure.

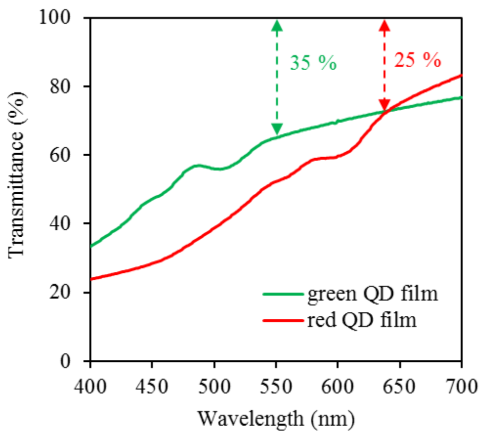

Figure S2. Transmittance spectra measured from the red and green QD film in vertical direction.
